# A Pan-Cancer Analysis Reveals CLEC5A as a Biomarker for Cancer Immunity and Prognosis

**DOI:** 10.3389/fimmu.2022.831542

**Published:** 2022-08-01

**Authors:** Rui Chen, Wantao Wu, Si-Yu Chen, Zheng-Zheng Liu, Zhi-Peng Wen, Jing Yu, Long-Bo Zhang, Zaoqu Liu, Jian Zhang, Peng Luo, Wen-Jing Zeng, Quan Cheng

**Affiliations:** ^1^ Department of Neurosurgery, The Affiliated Nanhua Hospital, Hengyang Medical School, University of South China, Hengyang, China; ^2^ Department of Neurosurgery, Xiangya Hospital, Central South University, Changsha, China; ^3^ Department of Oncology, Xiangya Hospital, Central South University, Changsha, China; ^4^ National Clinical Research Center for Geriatric Disorders, Xiangya Hospital, Central South University, Changsha, China; ^5^ Department of Pharmacy, Xiangya Hospital, Central South University, Changsha, China; ^6^ Department of Pharmacy, The Affiliated Hospital of Guizhou Medical University, Guizhou Medical University, Guiyang, China; ^7^ Department of Neurosurgery, and Department of Cellular & Molecular Physiology, Yale University School of Medicine, New Haven, CT, United States; ^8^ Department of Interventional Radiology, The First Affiliated Hospital of Zhengzhou University, Zhengzhou, China; ^9^ Department of Oncology, Zhujiang Hospital, Southern Medical University, Guangzhou, China

**Keywords:** pan-cancer, CLEC5A, prognostic biomarkers, cancer immune, immunotherapy

## Abstract

**Background:**

CLEC5A is a member of the C-type lectin superfamily. It can activate macrophages and lead to a series of immune-inflammation reactions. Previous studies reveal the role of CLEC5A in infection and inflammation diseases.

**Method:**

We acquire and analyze data from The Cancer Genome Atlas (TCGA) database, Genotype-Tissue Expression (GTEx) database, and other comprehensive databases *via* GSCALite, cBioPortal, and TIMER 2.0 platforms or software. Single-cell sequencing analysis was performed for quantifying the tumor microenvironment of several types of cancers.

**Results:**

*CLEC5A* is differentially expressed in a few cancer types, of which overexpression accompanies low overall survival of patients. DNA methylation mainly negatively correlates with *CLEC5A* expression. Moreover, *CLEC5A* is positively related to immune infiltration, including macrophages, cancer-associated fibroblasts (CAFs), and regulatory T cells (Tregs). Immune checkpoint genes are significantly associated with *CLEC5A* expression in diverse cancers. In addition, *CLEC5A* expression correlates with mismatch repair (MMR) in several cancers. Tumor mutation burden (TMB), microsatellite instability (MSI), and neoantigens show a positive association with *CLEC5A* expression in several cancers. Furthermore, *CLEC5A* in cancer correlates with signal transduction, the immune system, EMT, and apoptosis process. The drug sensitivity analysis screens out potential therapeutic agents associated with *CLEC5A* expression, including FR-180204, Tivozanib, OSI-930, Linifanib, AC220, VNLG/124, Bexarotene, omacetaxine mepesuccinate, narciclasine, leptomycin B, PHA-793887, LRRK2-IN-1, and CR-1-31B.

**Conclusion:**

CLEC5A overexpresses in multiple cancers in contrast to normal tissues, and high CLEC5A expression predicts poor prognosis of patients and immune infiltration. CLEC5A is a potential prognostic biomarker of diverse cancers and a target for anti-tumor therapy.

## Introduction

Cancer has the second mortality rate worldwide, and immunotherapy is one of the most promising cancer therapeutic strategies. Despite significant advances and successful application of immunotherapy across a wide range of human cancers, most patients had limited or no response to these therapies. Previous research indicated tumor mutation burden (TMB), the proportion of immune cell infiltration, and PD-L1/PD-L1 expression are widely recognized indicators for cancer immunotherapy response (1–4). However, these molecular markers have some limitations in predicting tumor immunotherapy response. Therefore, exploring more novel biomarkers to assess response to these immunotherapy approaches and helping develop a more effective treatment that ultimately improves clinical outcome for cancer patients is still urgently needed.

Human C‐type lectin domain family 5 member A (CLEC5A), also known as myeloid DAP12-associating lectin-1 (MDL-1), is a II-type transmembrane protein and only expresses on the myeloid lineages including macrophages, monocytes, neutrophiles, and dendritic cells (DCs) (5–7). As a pattern recognition receptor, CLEC5A transmits signals into cytoplasm through non-covalent binding to the adaptor protein DAP12 (8). Phosphorylation of DAP12 then initiates a Syk kinase-based signaling cascade resulting in macrophages activation and release of chemokines and proinflammation cytokines, including IL-6, TNF, CCL3, and CXCL8. No doubt that CLEC5A can trigger myeloid cell-related immune response and correlates with diverse infection and inflammatory diseases (9). CLEC5A has been proved to promote the production of high levels of pro-inflammatory cytokines and chemokines in flavivirus infections, especially dengue and Japanese encephalitis virus infections, and anti-CLEC5A mAb or CLEC5A inhibitors can reverse disease progression, suggesting that CLEC5A is a promising therapeutic target for flavivirus infections (10–13). In addition, similar in several autoimmune diseases, a high level of CLEC5A is found in active rheumatoid arthritis and CLEC5A activator increases proinflammation cytokines level (14). CLEC5A is closely related to the immune-inflammation process.

More recently, CLEC5A has emerged as a pivotal contributor to cancer development and progression (15–20). Aberrant high CLEC5A expression significantly correlates with decreased overall survival in high-grade serious ovarian cancer (HGSOC), gastric cancer, and glioma (18–21). In gastric cancer, *CLEC5A* silencing or knockdown results in decreased cell growth, colony-formation capability, and tumor volume and weight, which is consistent with the findings in GBM (17, 19). Mechanistically, CLEC5A correlates with the PI3K/Akt pathway in tumorigenesis (19). Moreover, CLEC5A expression in glioma positively correlates with immune score, but negatively correlates with tumor purity (21). Furthermore, CLEC5A expression in gliomas co-exists with more tumor-promoting leukocytes infiltration, especially M2 macrophages. However, the above findings are limited to finite cancer types, and more studies are essential to determine CLEC5A as a tumor prognostic biomarker and immunotherapy target.

This study systematically illustrated the CLEC5A profiles, including mRNA expression, methylation, mutation patterns, immune infiltration, correlation with signatures of interest, and patient’s prognosis. The function enrichment analysis also identified the possible pathways that CLEC5A participates in tumorigenesis. In addition, we identified potential chemotherapeutic drugs targeting CLEC5A. In general, this study contributes to a comprehensive understanding of the critical role of CLEC5A in tumorigenesis and tumor immunity and provides candidate drugs based on CLEC5A as anti-tumor therapeutic targets.

## Methods

### CLEC5A Expression Profiles

The gene expression data of *CLEC5A* in tumor and normal tissues and the patient’s prognosis information were obtained from The Cancer Genome Atlas (TCGA, http://cancergenome.nih.gov) database and genotype-tissue expression (GTEx, http://commonfund.nih.gov/GTEx/). To make gene expression data more comparable between samples, fragments per kilobase million (FPKM) values were converted to transcript per kilobase million (TPM) values, normalized by Log2 conversion. The expression difference of *CLEC5A* in cancer and normal tissues was visualized.

The gene expression profile data of *CLEC5A* expression in cancer cell lines were acquired from the Cancer Cell Line Encyclopedia (CCLE) platform (https://sites.broadinstitute.org/ccle/tools) (22). The *CLEC5A* mRNA expression profile in various cancer cell lines was achieved from the Human Protein Atlas (HPA, https://www.proteinatlas.org/) website (23, 24).

### Survival Analysis

Kaplan-Meier Plotter was utilized to analyze the correlation between *CLEC5A* expression and patient survival in different cancers (25). The results of univariate Cox regression were summarized and presented by a forest plot.

### Single Cell Sequencing Analysis

Single-cell sequencing datasets of BLCA, BRCA, CHOL, COAD, GBM, HNSCC, KIRC, LIHC, OV, SKCM, and STAD were acquired from the Gene Expression Omnibus (GEO) database (https://www.ncbi.nlm.nih.gov/geo/). A single-cell sequencing dataset of LUAD was downloaded from the NCBI BioProject (PRJNA591860), and a single-cell sequencing dataset of PAAD, CRA001160, was downloaded from the Genome Sequence Archive (GSA) database (https://ngdc.cncb.ac.cn/gsa/browse/CRA001160). The detailed information on these GEO datasets and R packages for data integration and quality control are shown in **Table 1**. After data processing, principal component analysis (PCA) was performed. Tumor cells were identified using the R package infercnv and copykat. Immune cells and stromal cells were annotated based on specific markers. The UMAP function was used for visual dimension reduction, and Vlnplot, Dimplot, and Featureplot were used for visualization of *CLEC5A* expression.

**Table 1:** The detailed information of these GEO datasets and R packages for data integration and quality control.

Cancer types	Datasets	R package
BLCA	GSE145137	
BRCA	GSE75688, GSE118389	Seurat
CHOL	GSE125449	
COAD	GSE81861	
GBM	GSE138794	
HNSCC	GSE103322	
KIRC	GSE121636, GSE171306	Seurat
LIHC	GSE125449	
OV	GSE118828	
SKCM	GSE72056	
STAD	GSE134520, GSE158631	harmony
LUAD	PRJNA591860	
PAAD	CRA001160	

### Methylation Profiles

GSCALite platform (http://bioinfo.life.hust.edu.cn/web/GSCALite/) was used to analyze differential methylation of *CLEC5A* in tumors and normal tissues across various TCGA cancer types (26). Subsequently, we used the same platform to explore the correlation between *CLEC5A* methylation and its expression in different cancers.

### Mutation Profiles

The copy number alteration (CNA) and mutation landscape of *CLEC5A* in pan-cancer was gained from cBioPortal (http://www.cbioportal.org) (27, 28). Thus, GSCALite was used to investigate the SNP frequencies and heterozygous/homozygous CNA of *CLEC5A*, *CD163*, *CD68*, *CD8A*, *CD8B*, and *MRC1* genes, as well as the correlation between CNA and mRNA expression of these genes.

### Immune-Related Characteristics

TIMER2.0 (http://timer.cistrome.org/) was utilized to analyze the relationship between *CLEC5A* expression and tumor-infiltration immune cells in tumors across various cancer types (29). Tumor purity was indirectly evaluated with the ESTIMATE score, and the abundance of immune and stromal components in tumors was assessed by the immune score and the stromal score, respectively. Subsequently, we evaluated the correlation between *CLEC5A* expression and the markers for immune cell subsets, including CD8+ T cell, macrophage, T cell regulatory (Tregs), and cancer-associated fibroblast (CAF). A P-value less than 0.05 indicated a significant correlation. We evaluated the relationship between *CLEC5A* gene expression and immune checkpoint (ICP), TMB, microsatellite instability (MSI), and neoantigens using the Pearson correlation analysis. Pearson correlation analysis was also implemented to evaluate the correlation between *CLEC5A* gene expression and MMR genes (*MLH1*, *MSH2*, *MSH6*, *PMS2*, and *EPCAM*) and DNA methyltransferases (*DNMT1*, *DNMT2*, *DNMT3A*, and *DNMT3B*).

### Functional Enrichment Analysis

The proteins interacting with CLEC5A protein were analyzed with the STRING database (https://string-db.org/). The OPEN TARGET platform (https://www.target-validation.org/) was used to identify CLEC5A related diseases network (30). Gene set enrichment analysis (GSEA) of the Kyoto Encyclopedia of Genes and Genomes (KEGG) terms and HALLMARK terms was performed to describe the biological functions and pathways related to CLEC5A. Gene set variation analysis (GSVA) of gene ontology (GO) was also achieved to describe the immune pathways related to CLEC5A. The top KEGG and HALLMARK terms were listed, which |NES|>1, NOM p <0.05, and FDR q <0.25 were set as the threshold for significantly enriched pathways. Moreover, the pathway activity module of the GSCALite platform was used to study the differences in genes expression between the activity groups (activation and inhibition) of cancer-related pathways in different cancer types.

### Immunofluorescence Staining

We obtained the tissue microarray from the Outdo Biotechcompany (HOrg-C110PT-01, Shanghai, China) and the ethics was approved. The primary antibodies were CLEC5A (Rabbit, Sigma-Aldrich, US), CD68 (Rabbit, AiFang biological, China), CD163 (Rabbit, Proteintech, China), CD8 (Mouse, Proteintech, China). The secondary antibody was horseradish peroxidase-conjugated secondary antibody incubation (GB23301, GB23303, Servicebio, China), and the tyramide signal amplification was TSA [FITC-TSA, CY3-TSA, 594-TSA, and 647-TSA (Servicebio, China)]. Multispectral images were analyzed, and positive cells were quantified at single-cell levels by Caseviewer (CV 2.3, CV 2.0) and Pannoramic viewer (PV 1.15.3) image analysis software.

### Immunotherapy Agents Response Prediction

*CLEC5A* expression levels were compared across tumor cell lines between pre- and post-cytokine treated samples and between pre- and post-ICB treated samples. The differences between groups were statistically evaluated by Wald test using DESeq2 (*FDR ≤ 0.05, **FDR ≤ 0.01, ***FDR ≤ 0.001), and the comparison results were summarized in boxplots. Tumor models included were: mammary cancer: 4T1, E0771, EMT6, T11, KPB25L, p53-2225L, p53-2336R; colorectal carcinoma: CT26, MC38; gastric adenocarcinoma: YTN16; head and neck squamous cell carcinoma: MOC22; hepatocellular carcinoma: BNL-MEA; lung carcinoma: LLC; melanoma: B16, YUMM1.7, D3UV2, D4M.3A.3; sarcoma: 402230.

### Drug Sensitivity Prediction

GSCALite integrates over 750 small molecule drugs from the Genomics of Drug Sensitivity in Cancer (GDSC)/Therapeutics Response Portal (CTRP). We conducted the drug sensitivity analysis *via* GSCALite platform to explore the correlation between drug sensitivity and *CLEC5A* expression. Spearman correlation analysis was used to evaluate the correlation. Positive correlation indicated that tumor cells with high *CLEC5A* expression are prone to drug resistance, while negative correlation suggested that tumor cells with high *CLEC5A* expression are sensitive to drugs.

### Statistical Analysis

The statistical significance of differences between groups was analyzed by Student’s t test, and the comparison among groups was analyzed by one-way ANOVA. *P*-value ≤ 0.05 indicated statistically significant.

## Results

### CLEC5A Is Abnormally Expressed in Human Pan-Cancer and Servers as a Prognostic Biomarker

**Figure 1:**
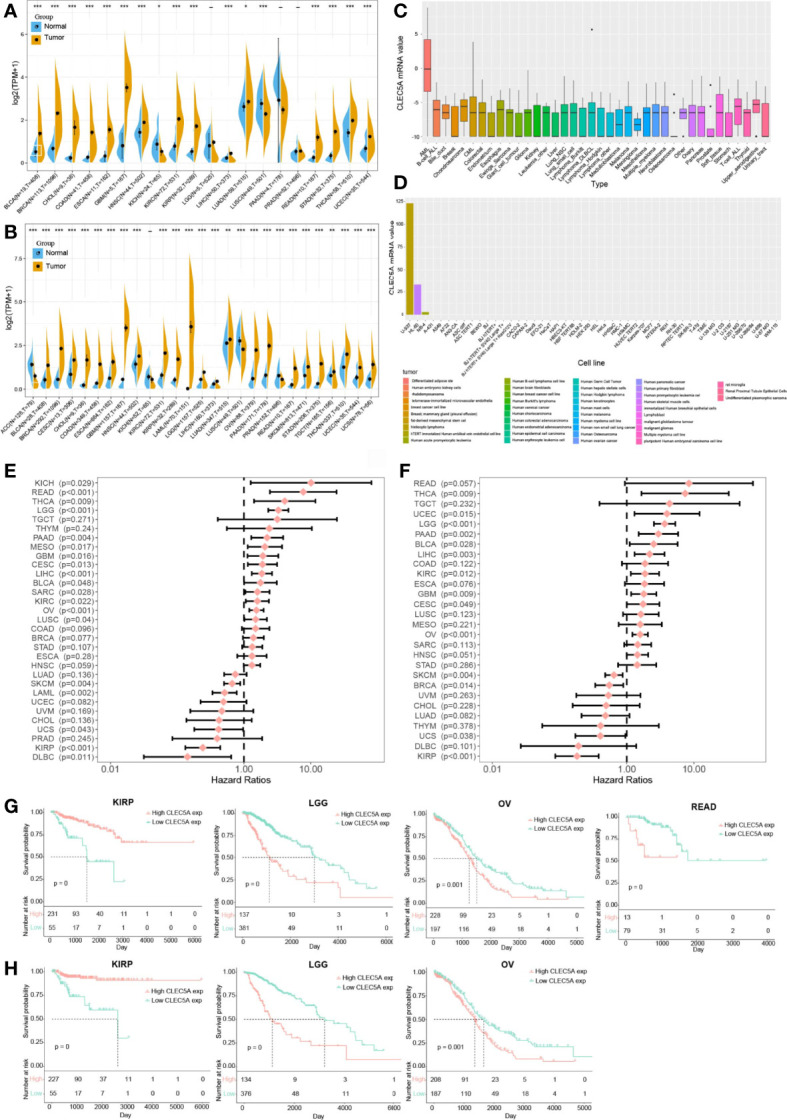
*CLEC5A* is abnormally expressed in human pan-cancer and serves as a prognostic biomarker. **(A)** *CLEC5A* expression in cancers and normal tissues from TCGA database. **(B)** *CLEC5A* expression in cancers and normal tissues combining the GTEx database and the TCGA database (*P < 0.05, **P < 0.01, *** *P*< 0.001). **(C)** *CLEC5A* expression in tumor cells from the CCLE database. **(D)** *CLEC5A* expression in tumor cells from the HPA website. **(E)** Forest plots showed the correlation between *CLEC5A* expression and patient’s overall survival (OS) in different cancers. **(F)** Forest plots showed the correlation between *CLEC5A* expression and patient’s disease-free survival (DFS) in different cancers. **(G)** Kaplan-Meier curves for patient’s OS stratified by different expression levels of *CLEC5A* in KIRP, LGG, OV, and READ. **(H)** Kaplan-Meier curves for patient’s DFS stratified by different expression levels of *CLEC5A* in KIRP, LGG, and OV.

First, we comprehensively analyzed the *CLEC5A* expression in tumors and paired normal tissues in different cancers with TCGA and GTEx gene expression data. The results showed that *CLEC5A* mRNA expression was significantly up-regulated in 23 tumors (BLCA (bladder urothelial carcinoma), BRCA (breast invasive carcinoma), CESC (cervical squamous cell carcinoma and endocervical adenocarcinoma), CHOL (cholangiocarcinoma), COAD (colon adenocarcinoma), ESCA (esophageal carcinoma), GBM (glioblastoma), HNSC (head and neck squamous cell cancer), KIRC (kidney renal clear cell carcinoma), KIRP (kidney renal papillary cell carcinoma), LAML (acute myeloid leukemia), LGG (lower grade glioma), LIHC (liver hepatocellular carcinoma), LUAD (lung adenocarcinoma), OV (ovarian serous cystadenocarcinoma), PAAD (pancreatic adenocarcinoma), READ (rectum adenocarcinoma), SKCM (skin cutaneous melanoma), STAD (stomach adenocarcinoma), TGCT (testicular germ cell tumors), THCA (thyroid carcinoma), UCEC (uterine corpus endometrial carcinoma), and UCS (uterine carcinosarcoma)) and down-regulated in 4 tumors [ACC (adrenocortical carcinoma), KICH (kidney chromophobe), LUSC (lung squamous cell carcinoma), and PRAD (prostate adenocarcinoma)] (**Figures 1A, B**). In human tissues, *CLEC5A* mRNA expression was enriched in AML, B-cell, and T-cell of ALL, bile duct, and other tissues (**Figure 1C**). Moreover, *CLEC5A* mRNA expression was highly expressed in immortal tumor cell lines, such as U937, HL-60, and NB-4 (**Figure 1D**). Therefore, CLEC5A differential expression in most cancer types indicates that CLEC5A may play a key role in carcinogenesis.

We next explored the correlation between CLEC5A expression and the prognosis of patients with pan-cancer. By univariate Cox regression analysis of 32 cancer types, we found that high *CLEC5A* expression indicated poor OS and DFS in patients with THCA, LGG, PAAD, GBM, CESC, LICH, KIRC, and OV but predicted better OS and DFS in patients with SKCM, UCS, and KIRP (**Figures 1E, F** **)**. In the TCGA dataset, *CLEC5A* high expression was correlated with poor OS in patients with LGG, OV, and READ, and *CLEC5A* high expression was also associated with poor DFS in patients with LGG and OV (**Figures 1G, H** **)**. In the TCGA dataset, CLEC5A high expression was correlated with better OS and DFS in patients with KIRP. Moreover, the expression of *CLEC5A* was also negatively associated with the survival of patients with LIHC, LUAD, and PAAD in GSE39582, GSE13213, and GSE57495, respectively (**Supplementary Figure S1**).

### Single Cells Analysis of CLEC5A in Human Cancers

**Figure 2:**
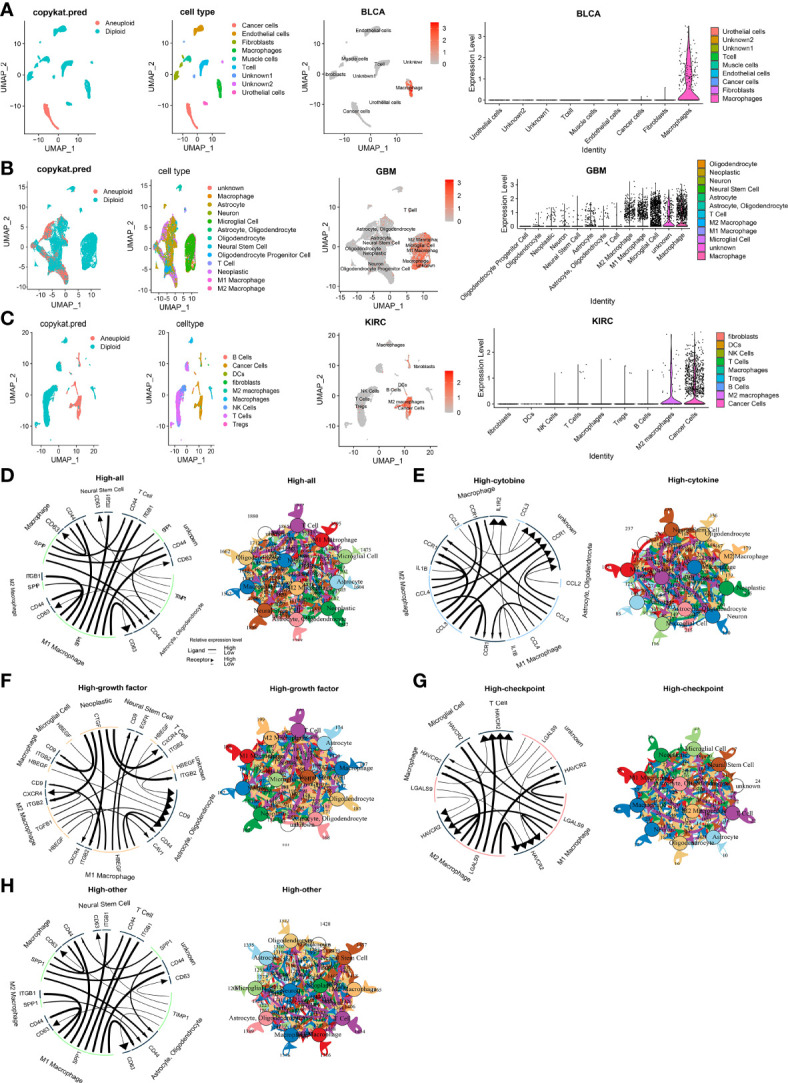
Single cells analysis of *CLEC5A* in human cancers. **(A–C)** Composition and distribution of single cells and CLEC5A expression in single cells in BLCA, GBM, KIRC. Each cell type is ranked by its average expression value. **(D)** The overall cellular communication pattern regarding high CLEC5A expression in GBM. **(E)** The cytokine-associated cellular communication pattern regarding CLEC5A expression in GBM. **(F)** The growth factor-associated cellular communication pattern regarding CLEC5A expression in GBM. **(G)** The checkpoint-associated cellular communication pattern regarding CLEC5A expression in GBM. **(H)** The other-associated cellular communication pattern regarding CLEC5A expression in GBM.

To clarify *CLEC5A* mRNA expression in different cell types in pan-cancer, we obtained single-cell RNA sequencing data of BLCA, BRCA, CHOL, COAD, GBM, HNSCC, KIRC, LIHC, LUAD, OV, PAAD, SKCM, and STAD for analysis. The results showed that macrophages exhibited higher *CLEC5A* expression than other cell types in BLCA, COAD, GBM, PAAD, and SKCM. The *CLEC5A* expression in M2 macrophages in BRCA and HNSCC was higher than that in other cell types, while the *CLEC5A* expression in M1 macrophages and cancer cells in OV was higher than that in different types **(** **Figures 2A–C** and **Supplementary Figures 2, 3** **)**. In addition, the ligand-receptor interactions in cells with high CLEC5A expression and 4 categories of intercellular communication(cytokine, growth factor, checkpoint, and other) were identified (**Figures 2D–H**). Obviously, strong interactions among neoplastic, T cells, M1 macrophages, and M2 macrophages were observed in growth factor analysis (**Figure 2F**). These results indicated that CLEC5A widely existed in tumor cells and macrophages in the tumor microenvironment, and suggested that CLEC5A may influence tumor progression and prognosis by participating in the tumor microenvironment and tumor immunity regulation.

### The Methylation Analysis of *CLEC5A* in Human Cancers

**Figure 3:**
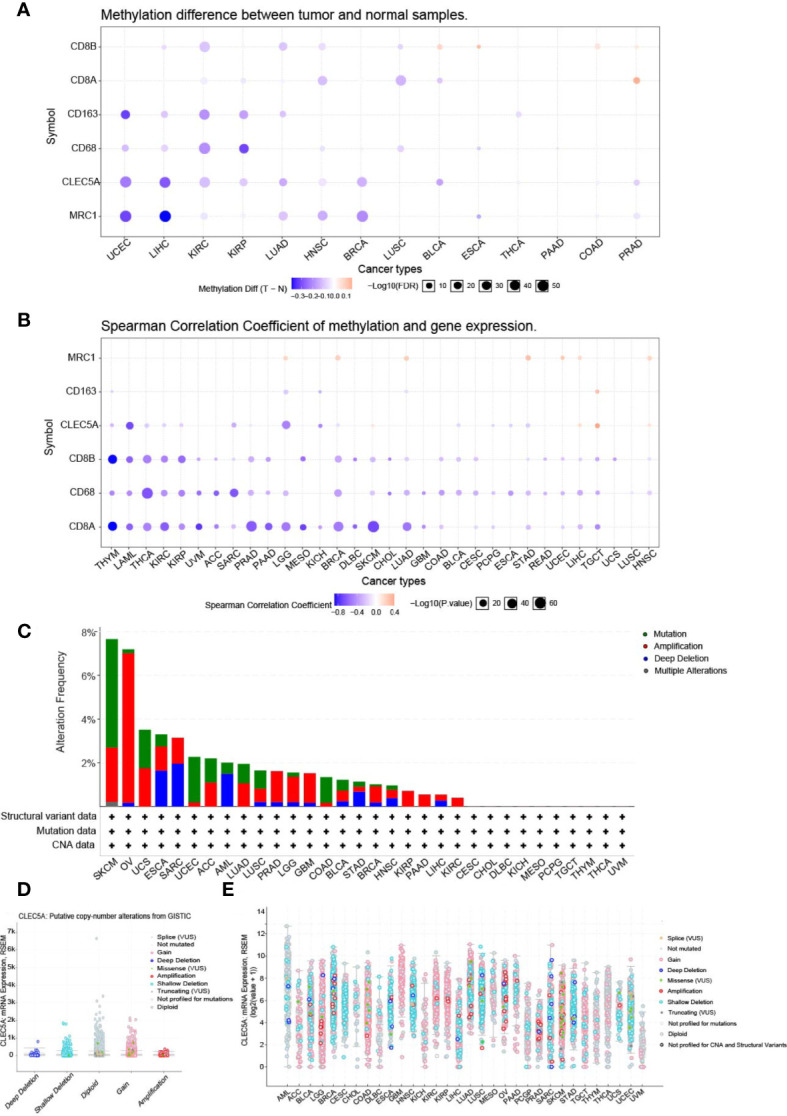
The methylation analysis and mutation landscape of CLEC5A in human cancers. **(A)** DNA methylation differences of *CLEC5A* between tumors and normal tissues in various cancers with GSCALite platform. **(B)** Correlation between the DNA methylation and mRNA expression of *CLEC5A* in 32 cancer types. **(C)** *CLEC5A* alteration (mutation and CNAs) frequency in 32 TCGA cancer types. **(D, E)** Correlation between the putative copy-number alteration of *CLEC5A* and its expression in tumors.

Research has confirmed that DNA methylation is closely related to changes in gene expression in cancers (31). Thus, the DNA methylation differences of *CLEC5A* and immune-related gene (*MRC1, CD163, CD8A, CD8B, CD68*) between tumors and normal tissues in various cancers were evaluated with the GSCALite platform. The results indicated that *CLEC5A* methylation was significantly down-regulated in UCEC, LIHC, KIRC, KIRP (kidney renal papillary cell carcinoma), LUAD, HNSC, BRCA, BLCA, and PRAD (prostate adenocarcinoma) (**Figure 3A**). Next, we evaluated the correlation between the DNA methylation and mRNA expression of *CLEC5A* and immune-related gene (*MRC1, CD163, CD8A, CD8B, CD68*) in pan-cancer. The results exhibited that the *CLEC5A* mRNA expression was mainly negatively correlated with its DNA methylation in most tumors but only positively correlated with its DNA methylation in only some tumors, such as SKCM (cutaneous skin melanoma), TGCT (testicular germ cell tumors), LIHC and HNSC (**Figure 3B**). Moreover, except for MRC1, macrophage biomarker genes (*CD163, CD68*) and T cells biomarker genes (*CD8A, CD8B*) also mainly showed a negative correlation with their DNA methylation (**Figure 3B**).

### The Mutation Landscape of *CLEC5A* in Human Cancers

To study the mutation landscape of *CLEC5A* in human cancers, the cBioPortal tool was utilized to analyze the *CLEC5A* alteration (mutation and CNAs) frequency in 32 TCGA cancers types. The results showed that the alteration frequency of *CLEC5A* varies with tumor type. As shown in **Figure 3C**, *CLEC5A* was found to have a high alteration frequency of nearly 8% in both SKCM and OV, but no related alteration was found in the other 10 cancer types (CSCC, CHOL, DLBC, KICH, MESO, PCPG, TGCT, THYM, THCA, and UVM). Moreover, in the four alteration types (mutation, amplification, deletion, and multiple changes) of *CLEC5A*, copy-number alteration (CNA, including deletion and amplification) frequency was the highest, followed by mutation and considerable alteration. Then, we explored the correlation between the putative CNA of *CLEC5A* and its gene expression in tumors. The putative CNA of CLEC5A in pan-cancer were shown in (**Figures 3D, E** **)**.

### *CLEC5A* CNVs, SNV, and Alternative Splicing in Human Cancers

**Figure 4:**
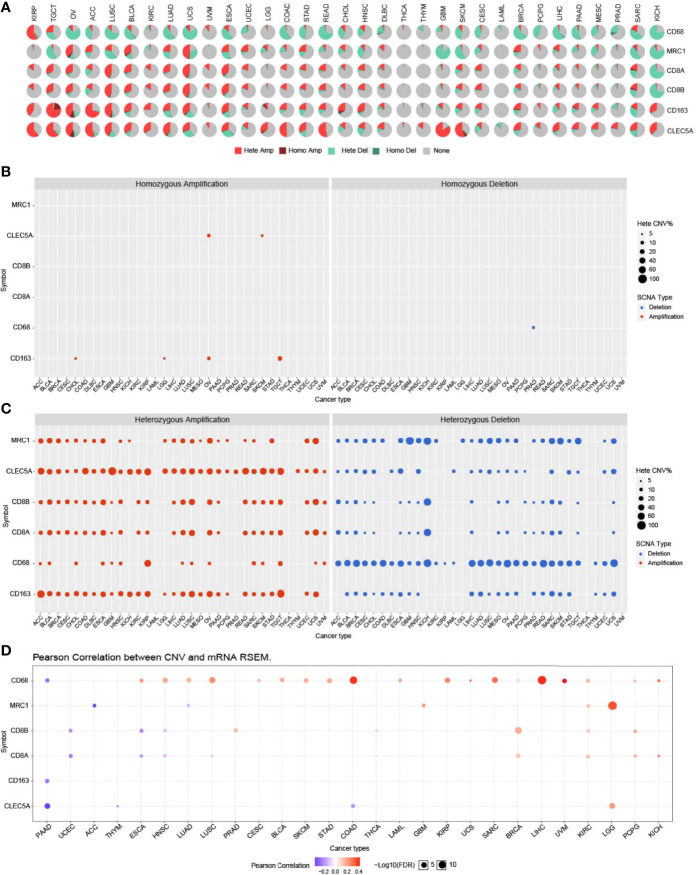
*CLEC5A* CNVs in human cancers. **(A)** CNV profiles of *CLEC5A*, *CD68, CD163, MRC1, CD8A*, and *CD8B* in 33 cancer types with the GSCALite platform. Hete Amp, heterozygous amplification; Hete Del, heterozygous deletion; Homo Amp, homozygous amplification; Homo Del, homozygous deletion. **(B)** In pan-cancer, homozygous amplification or deletion CNV of *CLEC5A*, *CD68, CD163, MRC1, CD8A,* and *CD8B*. Blue bubbles represent deletion and red bubbles represent amplification, whereas the size of bubbles refers to the percentage of CNV. **(C)** In pan-cancer, heterozygous amplification or deletion CNV of *CLEC5A*, *CD68, CD163, MRC1, CD8A,* and *CD8B*. **(D)** Correlation between CNV and mRNA expression of *CLEC5A*, *CD68, CD163, MRC1, CD8A,* and *CD8B* in pan-cancer. Blue bubbles represent a negative correlation, whereas red bubbles represent a positive correlation, and the darker the color, the higher the correlation. The size of each bubble means statistical significance.

Copy number variation (CNV) module of the GSCALite platform provided heterozygous and homozygous CNV profiles in 33 cancer types. The pie chart showed that the heterozygous CNVs of all 6 genes (*CD68*, *MRC1*, *CD8A*, *CD8B*, *CD163*, and *CLEC5A*) was more frequent in various cancers (**Figure 4A**). *CLEC5A* homozygous amplification only existed in OV and SKCM, and no homozygous deletion was found (**Figure 4B**). In most tumors, *CLEC5A* heterozygous amplification was standard, and CD68 heterozygous deletion was dominant (**Figure 4C**). Additionally, the correlation between CNV and gene expression was investigated. The result showed that *CLEC5A* expression was positively correlated with CNV only in LGG and negatively correlated with CNV in PAAD, THYM, and COAD. In contrast, CD68 expression was strongly and positively correlated with CNV in most cancer types (**Figure 4D**). Therefore, the CNV seems to have limited influence on *CLEC5A* expression.

Single nucleotide variation (SNV) is a common gene mutation associated with gene expression or function. GSCALite was utilized to identify the SNV frequency of all 6 genes (*CD68*, *MRC1*, *CD8A*, *CD8B*, *CD163*, and *CLEC5A*) in various cancers. As the waterfall plots exhibited, the SNV frequency of *CD163* was 68%, and that of *CLEC5A* was 12%, with missense mutation accounting for the majority (**Supplementary Figure S4A**). *CLEC5A* SNV frequency was higher in SKCM, COAD, BRCA, LUAD and LGG. Besides, we obtained a mutation pattern of *CLEC5A* in various cancers using the cBioportal tools, and the results coincided with the waterfall plots obtained by GSCALite (**Supplementary Figure S4B**).

Alternative splicing represents a robust method regulate gene expression and protein complexity at the mRNA level. A mutation frequently occurs in *CLEC5A*, and it is located at alternative splicing sites; we used OncoSplicing to explore the association between *CLEC5A* and alternative splicing (32). In **Supplementary Figure S5A**, PanPlot displayed the percent spliced in (PSI) distribution of alternative splicing events across different TCGA cancers and GTEx tissues. Differential alternative splicing analyses showed a significant difference in alternative splicing events between TCGA tumor tissue and GTEx normal tissue in LUSC and LUAD but no significant differences in alternative splicing events between TCGA tumor tissue and adjacent normal tissue (**Supplementary Figure S5B**). In addition, the PanCox plot showed that based on the median and optimal PSI cutoffs, the PSI distribution of CLEC5A alternative splicing events was significantly negatively correlated with progression-free survival (PFS) in HNSC, KIRC, BRCA and positively associated with PFS in PAAD and LUAD, while negatively correlated with OS in LUSC, LUAD, HNSC and positively associated with OS in PAAD (**Supplementary Figures 5C, D**), indicating the existence of survival-associated CLEC5A alternative splicing events.

### CLEC5A Expression Correlates With Immune Cells Infiltration in Human Cancers

To identify the role of CLEC5A in the immune infiltration process, we evaluated the correlation between CLEC5A expression and immune and stromal components and tumor purity in pan-cancer. The ESTIMATE score indirectly reflected the tumor purity, and immune and stromal scores represented the immune and stromal components that indirectly reflected tumor purity. **Supplementary Figures 6A–C** exhibited the top 6 cancer types with a high immune score, ESTIMATE scores, and stromal scores. *CLEC5A* expression was positively correlated with an immune score in BLCA, COAD, KICH, LGG, OV, and THCA. Meanwhile, *CLEC5A* expression was positively correlated with stromal components abundance in COAD, DLBC, KICH, LUSC, PAAD, and READ. In addition, ESTIMATE score showed a positive correlation with *CLEC5A* expression in BLCA, COAD, KICH, LUSC, OV, and READ, with r^2^ > 0.6. Tumor progression is intimately related to the alteration of immune infiltration and TME composition in cancers. The above results indicated that *CLEC5A* was highly involved in immune infiltration and diverse TME components formation in the above cancers.

**Figure 5:**
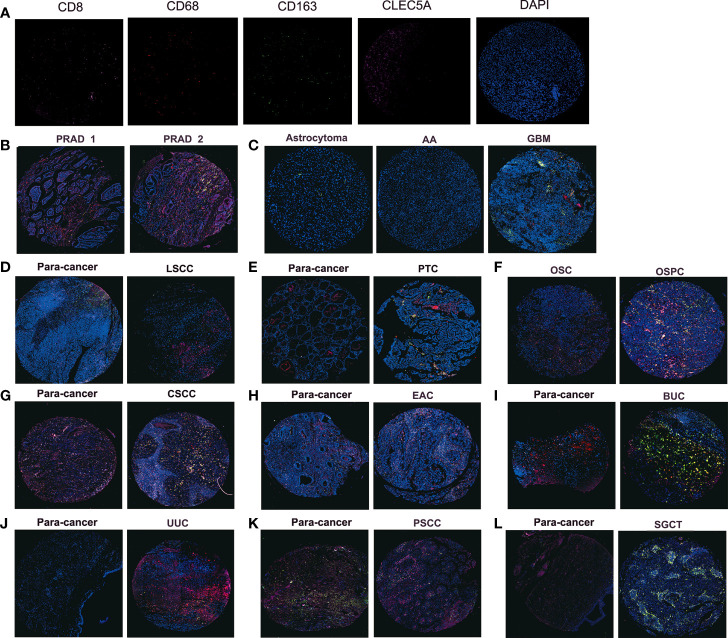
Immunofluorescence staining detects the protein expression of CLEC5A, CD8, CD68, and CD163 in tumor tissue chips. **(A)** Representative immunofluorescence staining image of CD8 (pink), CD68 (red), CD163 (green), CLEC5A (rose), and DAPI (blue) in positive control tissues. **(B)** The protein expression of CLEC5A, CD8, CD68, and CD163 in PRAD tissues with different Gleason scores, PRAD1: Gleason score=2, PRAD1: Gleason score=3. **(C)** The protein expression of CLEC5A, CD8, CD68, and CD163 in astrocytoma, AA, and GBM. **(D, E)** The protein expression of CLEC5A, CD8, CD68, and CD163 in LSCC, PTC and para-cancer tissues. **(F)** The protein expression of CLEC5A, CD8, CD68, and CD163 in OSC and OSPC. **(G–L)** The protein expression of CLEC5A, CD8, CD68, and CD163 in CSCC, EAC, BUC, UUC, PSCC, SGCT, and para-cancer tissues. PRAD, prostate adenocarcinoma; AA, anaplastic astrocytoma; GBM, glioblastoma; LSCC, laryngeal squamous cell carcinoma; PTC, papillary thyroid carcinoma; OSC, ovarian serous adenocarcinoma; OSPC, ovarian serous papillary adenocarcinoma; CSCC, cervical squamous cell carcinoma; EAC, endometrioid adenocarcinoma; BUC, bladder urothelial carcinoma; UUC, ureteral urothelial carcinoma; PSCC, penis squamous cell carcinoma; SGCT, seminoma.

Furthermore, we explored the relationship between CLEC5A expression and specific immune cells infiltration in human cancer. The results showed that *CLEC5A* expression was positively correlated with the immune infiltration levels of macrophages, cancer associated fibroblast, and Tregs, and negatively correlated with MDSC abundance (**Supplementary Figure S6D**). Notably, we performed immunofluorescence staining on tumor tissue chips, and CD8 positive T cells were labeled with pink fluorescence, CD68 positive macrophages were labeled with red fluorescence, CD163 positive macrophages were labeled with green fluorescence, CLEC5A was labeled with rose-red fluorescence, and DAPI was used to counterstain the nucleus (**Figure 5A–L**). In PRAD (prostate adenocarcinoma) tissues, CLEC5A expression was increased to accompany the increase of the Gleason score and the infiltration of macrophages, M2-type macrophages, and T cells also increased (**Figure 5B**). In GBM, CLEC5A was higher than that in astrocytoma and anaplastic astrocytoma, accompanied by an increase in M2-type macrophage infiltration (**Figure 5C**). CLEC5A expression was significantly higher in LSCC (laryngeal squamous cell carcinoma), PTC (papillary thyroid carcinoma) and UUC (ureteral urothelial carcinoma)than in para-cancer tissues, and was positively correlated with the infiltration proportion of macrophages (**Figures 5D, E, J**). Compared with OSC (ovarian serous adenocarcinoma), CLEC5A expression was higher in OSPC (ovarian serous papillary adenocarcinoma) and was significantly correlated with the increased infiltration of macrophages, M2-type macrophages, and T cell (**Figure 5F**). These results suggested that CLEC5A may be involved in the immune infiltration process and play an essential role in the immune-tumor interaction.

### CLEC5A Expression Is Related To Immune Checkpoints, Mismatch Repair, Tumor Mutational Burden, Microsatellite Instability, and Neoantigen in Human Cancers

**Figure 6:**
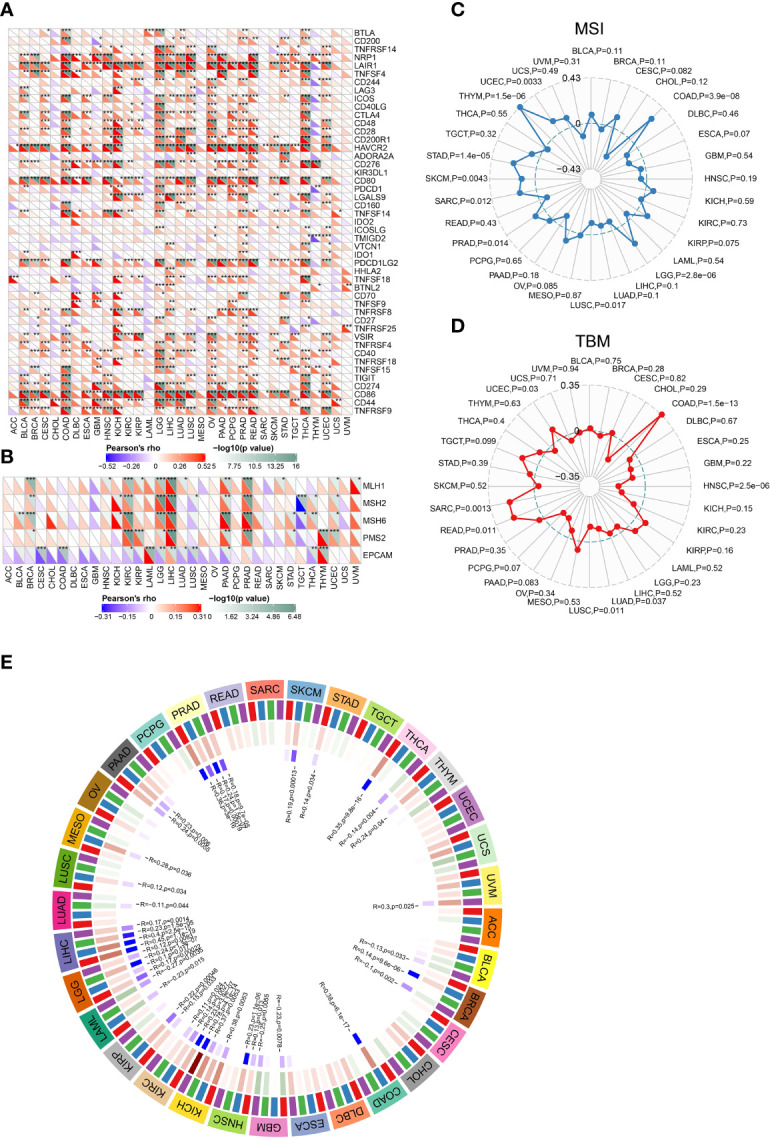
Correlations between *CLEC5A* expression and ICPs, MMRs, MSI, and TMB. **(A)** Correlation between *CLEC5A* expression and ICP gene levels in pan-cancer. **(B)** Heatmap of correlation between *CLEC5A* expression and MMR signatures (MLH1, MSH2, MSH6, PMS2, and EPCAM). *P < 0.05, **P < 0.01, ***P < 0.001. **(C)** Radar map of correlation between *CLEC5A* expression and MSI. **(D)** Radar map of correlation between *CLEC5A* expression and TMB. **(E)** Pearson correlation analysis of *CLEC5A* expression and DNA methyltransferases in pan-cancer.

Increased studies have demonstrated that immune checkpoints (ICPs) play important roles in tumor infiltration and immunotherapy (33). To determine the possibility of CLEC5A as a tumor immunotherapy target, the relationship between CLEC5A expression and ICPs expression in pan-cancer was explored. As shown in **Figure 6A**, *CLEC5A* expression in most cancers was positively correlated with the expression of ICPs, especially in LGG, OV, PAAD, and THCA and more than 30 immune checkpoints were positively correlated with *CLEC5A* expression. The strong correlation between CLEC5A and immune checkpoints suggested that CLEC5A might be an ideal tumor immunotherapeutic target and play certain roles in tumor immunotherapy response and outcome.

DNA mismatch repair (MMR) is essential to DNA replication and genetic recombination (34). MMR defects are common in tumors and can lead to the appearance of MSI, which in turn aggravates TMB (35, 36). At first, we studied the correlation between CLEC5A expression and MMR gene signature and found that *CLEC5A* expression was positively correlated with the expression of *MLH1*, *MSH2*, *MSH6*, and *PMS2* in KIRC, BRCA, LGG, LIHC, PADD, and PRAD, and negatively correlated with EPCAM in CESC, COAD, KIRC, LGG, LUAD, LUSC, PADD, and THCA (**Figure 6B**). MSI, TMB, and neoantigens are all intimately related to tumor initiation and progression, and they can independently predict the efficacy of tumor immunotherapy. Next, we conducted the correlation analysis between *CLEC5A* expression and MSI, TBM, and neoantigens. The results showed that *CLEC5A* expression was significantly positively correlated with MSI in LGG, COAD, PRAD, UCEC, THYM, SARC, STAD, and SKCM, but negatively correlated with MSI in LUSC (**Figure 6C**). In addition, positive correlation was observed between *CLEC5A* expression and TMB in COAD, READ, SARC, and UCEC, whereas negative correlation was found between *CLEC5A* expression and TMB in HNSC, LUAD, and LUSC (**Figure 6D**). As for neoantigen, it was positively correlated with *CLEC5A* expression in KIRP, UCEC, COAD, and STAD but negatively correlated with neoantigen in HNSC (**Supplementary Figure S7**). These results indicated that CLEC5A expression might influence tumor initiation and play a vital role in predicting the efficacy of tumor immunotherapy.

Numerous studies have found that abnormal DNA methylation catalyzed by DNMTs is involved in the occurrence and progression of various cancers (37, 38). We investigated the correlation between CLEC5A expression and four essential DNMTs expressions in pan-cancer. As shown in **Figure 6E**, *CLEC5A* expression was significantly correlated with the expression of *DNMT* signatures in more than 20 tumors. The result suggested CLEC5A may respond to oncogenesis by influencing DNA methylation.

### Enrichment Analysis of *CLEC5A*-Related Biological Functions in Pan-Cancer

**Figure 7:**
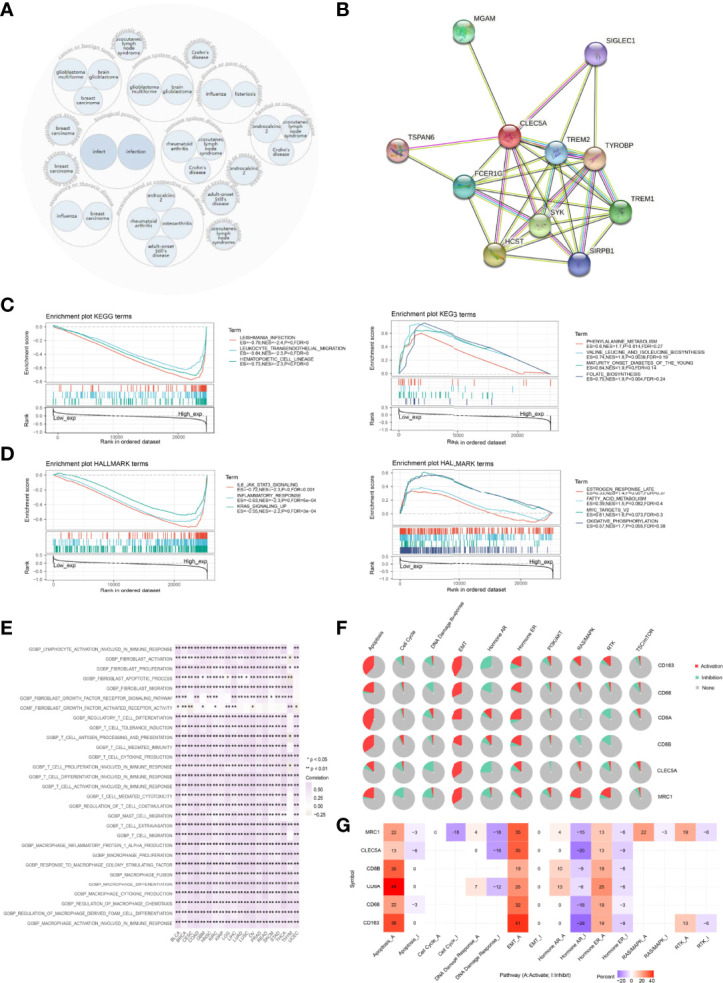
Functional annotation of *CLEC5A* in pan-cancer. **(A)** *CLEC5A* related diseases network. **(B)** *CLEC5A*-binding proteins were obtained using the STRING tool. **(C)** KEGG enrichment analysis related to *CLEC5A* expression using GSEA. **(D)** HALLMARK enrichment analysis related to *CLEC5A* expression using GSEA. **(E)** GSVA of GO terms associated with *CLEC5A* expression in pan-cancer. **(F)** The pathway activity module of GSCALite was used to investigate the impact of the gene set (*CLEC5A, CD68, CD163, MRC1, CD8A,* and *CD8B*) on tumor pathway activity in pan-cancer. **(G)** The percentage of cancers in which mRNA expression of 6 specific genes (*CLEC5A, CD68, CD163, MRC1, CD8A,* and *CD8B*) had a potential impact on pathway activity.

The OPENTARGET platform was used to identify *CLEC5A*-related diseases network. The results showed that *CLEC5A* is highly associated with nervous system diseases, immune system diseases, infectious diseases, and musculoskeletal or musculoskeletal or connective tissue diseases (**Figure 7A**). To study the molecular mechanism of *CLEC5A* in tumorigenesis, we used the STRING tool to predict *CLEC5A*-binding proteins and conducted functional enrichment analysis for *CLEC5A*-related genes using GSEA. As shown in **Figure 7B**, we obtained 10 CLEC5A-binding proteins, including TREM2, SIGLEC1, FCER1G, SYK, HCST, SIRPB1, TREM1, TYROBP, TSPAN6, and MGAM. KEGG enrichment analysis showed that the top three enriched terms related to the CLEC5A expression included Leishmania infection, leukocyte trans-endothelial migration, and hematopoietic cell linage (**Figure 7C**). KEGG enrichment analysis revealed that *CLEC5A*-related partners in pan-cancer were involved in B cell receptor signaling, T cell receptor signaling, and JAK-STAT signaling (**Supplementary Figure S8A**). HALLMARK enrichment analysis found that the top three enriched terms related to the *CLEC5A* expression included IL6-JAK-STAT3 signaling, inflammatory response, and KRAS signaling up (**Figure 7D** and **Supplementary Figure S8B**). Moreover, GSVA of GO terms related to *CLEC5A* expression was performed in pan-cancer (**Figure 7E**). *CLEC5A* significantly correlated with activation and migration of immune infiltrating cells such as T cells, macrophages, and mast cells. All these suggested that *CLEC5A* participates in tumorigenesis by impacting the immune response.

Then, GSCALite was utilized to investigate the impact of the gene set on tumor pathway activity. The pie chart showed that *CLEC5A* had an activation effect on the EMT pathway and an inhibitory effect on DNA damage response and the hormone AR pathway. All 6 genes could activate EMT, apoptosis, and hormone ER pathway and inhibit the hormone AR pathway (**Figure 7F**). In addition, the percentage of cancers in which mRNA expression of 6 specific genes (*CD68*, *MRC1*, *CD8A*, *CD8B*, *CD163*, and *CLEC5A*) had a potential impact on pathway activity were also summarized in **Figure 7G**. *CLEC5A* mediated activation of the EMT signaling pathway in 35% of cancers, while *CLEC5A* mediated inhibition of DNA damage response and hormone AR pathway in 18% and 25% of cancers, respectively.

### Immunotherapy Agent Response Associated With *CLEC5A* Expression

**Figure 8:**
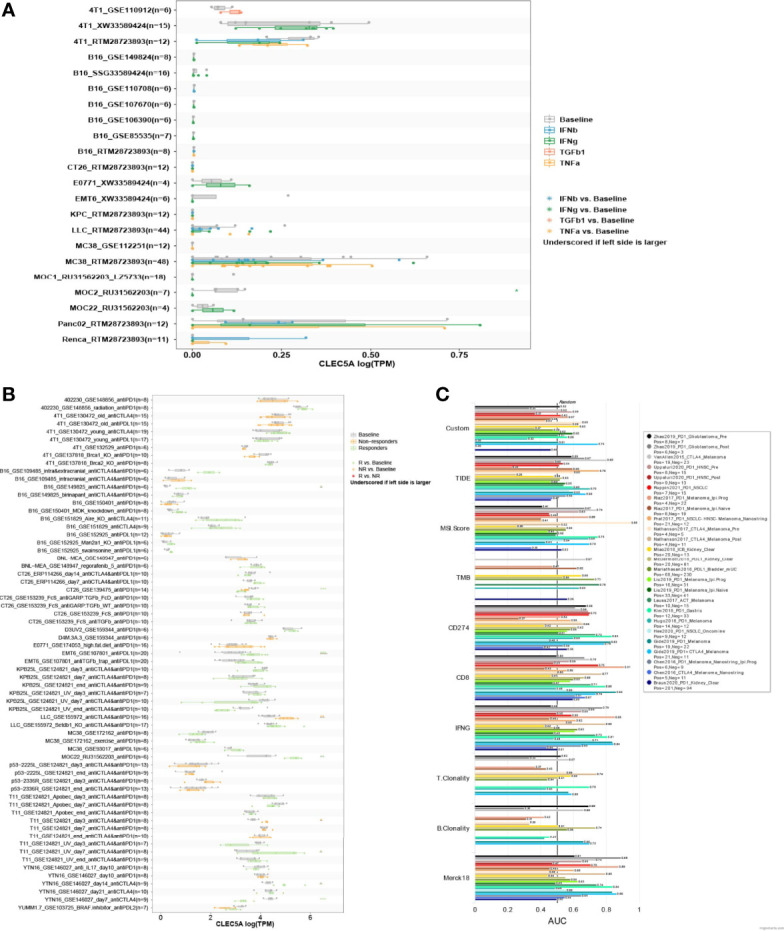
Correlation analysis of *CLEC5A* expression and minor molecule/drug sensitivity (IC50). **(A)** *CLEC5A* expression level between pre- and post-cytokine treatment across tumor cell lines. Cytokine treatments included in this module: IFNγ, IFNβ, TNFα, and TGFβ1. **(B)** *CLEC5A* expression level between pre- and post-ICB treatment and responders and non-responders across different tumor models. ICB treatments included in this module: anti-PD1, anti-PDL1, anti-PDL2, and anti-CTLA4. **(C)** The relationship between *CLEC5A* expression levels and the response outcomes and OS of human immunotherapy cohort.

To investigate the correlation between *CLEC5A* expression in tumor cells and their sensitivity to immunotherapy agents, *CLEC5A* expression levels across tumor cell lines were compared between pre- and post-cytokine-treated samples. As shown in **Figure 8A**, IFNγ treatment significantly down-regulated *CLEC5A* expression in MOC2 cells, while IFNγ, IFNβ, TNFα, and TGFb1 treatment did not change substantially *CLEC5A* expression in 4T1, B16, CT26, E0771, EMT6, KPC, LLC, MC38, MOC1, MOC22, Panc02, and Renca cells. We then compared the *CLEC5A* expression level across different tumor cell lines between pre- and post-ICB treatment and responders and non-responders. CLEC5A expression was significantly increased in B16 cells and T11 cells that responded to anti-CTLA4 and anti-PD-1 treatment. In addition, *CLEC5A* expression was increased in MOC22 cells that responded to anti-PD-1 therapy, and *CLEC5A* expression was increased in EMT6 that responded to anti-PDL-1 treatment. In contrast, *CLEC5A* expression was significantly decreased in CT26 cells that responded to anti-PD-1 therapy (**Figure 8B**).

Next, we evaluated the correlation between *CLEC5A* expression and the response outcomes and OS of the human immunotherapy cohort. The results showed that CLEC5A alone had an AUC of > 0.5 in 14 of the 25 immunotherapy cohorts (**Figure 8C**). Moreover, *CLEC5A* showed a higher predictive value than TMB, T. Clonality, and B. However, *CLEC5A* had lower predictive value scores than the CD274 score (AUC > 0.5 in 21 immunotherapy cohorts), CD8 score (AUC > 0.5 in 18 immunotherapy cohorts), IFNG score (AUC > 0.5 in 17 immunotherapy cohorts), and Merck 18 score (AUC > 0.5 in 18 immunotherapy cohorts). Above all, CLEC5A expression can function as an effective biomarker for predicting immunotherapy response.

### Identify Potential Therapeutic Agents Associated With CLEC5A Expression

According to the gene expression, survival analysis, mutation analysis, and immune infiltration analysis of *CLEC5A* in pan-cancer, we confirmed that *CLEC5A* might play an essential role in tumorigenesis and tumor immunity, suggesting its enormous potential as a tumor therapeutic target. To identify potential therapeutic agents associated with CLEC5A expression, we conducted a Spearman correlation analysis between *CLEC5A* expression and drug sensitivity (IC50) *via* the GSCALite website. The results indicated that *CLEC5A* expression was negatively correlated with IC50 of small molecule drugs such as FR-180204, Tivozanib, OSII-930, Linifanib, AC220, VNLG/124, Bexarotene, Omacetaxine mepesuccinate, narciclasine, leptomycin B, PHA-793887, LRRK2-IN-1, and CR-1-31B, but positively associated with IC50 of JW-55 (**Supplementary Figure S9**).

## Discussion

Pan-cancer analysis can reveal the similarities and differences of tumors, which provide theoretical support for cancer prevention, therapeutic target design, and potential therapeutic drug screening (39). Emerging publications indicated that *CLEC5A* plays a vital role in immune-inflammation response covering macrophage activation, proinflammation cytokines releasing, and neutrophil extracellular trap (NET), and *CLEC5A* high expression appears in diverse diseases, especially tumors (40–43). Although *CLEC5A* has been studied in glioma, gastric cancer, breast cancer, and ovarian cancer (15–20), its roles in pan-cancer remain unclear. In the present study, we performed a comprehensive bioinformatics analysis to reveal the abnormal expression, DNA methylation, alteration, and immune landscape in pan-cancer, which vary significantly among different cancers. We found that gene mutations and methylation levels of *CLEC5A* can lead to abnormally high *CLEC5A* expression, which is significantly associated with increased immune infiltration of macrophages, CAF, and Treg and poor prognosis of various cancers. In addition, a significant association exists between *CLEC5A* expression and ICP, MMR, TBM, MSI, and neoantigen markers in multiple cancers as well. Furthermore, *CLEC5A*’s high expression can also serve as a biomarker for immunotherapy response and exhibit low sensitivity to small molecule drugs.

Previous studies indicated that CLEC5A is overexpressed in some cancers and involves tumorigenesis and progression (15–20). Consistent with previous studies, our results also showed that *CLEC5A* was overexpressed in glioma and breast cancer compared with their regular counterparts. In addition, *CLEC5A* mRNA expression was up-regulated in 22 other cancer types and down-regulated in only 4 tumors (ACC, KICH, LUSC, and PRAD). Both genetic mutation and epigenetic modification can induce abnormal gene expression during tumorigenesis. In this study, *CLEC5A* gene alteration occurs in most cancer types, predominately amplification, followed by mutation. *CLEC5A* expression positively correlates with CNV in LGG but negatively correlates with CNV in PAAD and COAD. Besides, we found that the SNV frequency of *CLEC5A* reaches 12%, of which missense mutations were the most common, mainly occurring in SKCM, COAD, UCEC, LUAD and LUSC. Moreover, our results found that high *CLEC5A* expression indicated poor OS and DFS in patients with THCA, LGG, PAAD, GBM, CESC, LICH, KIRC, and OV but predicted better OS and RFS in patients with SKCM, UCS, and KIRP.

MMR is an essential system for maintaining stable DNA replication, and MMR dysfunction leads to the accumulation of mutation resulting in increased MSI rising, which is conducive to tumor initiation (34–36). In this study, *CLEC5A* expression was correlated with five MMR genes expression in pan-cancer, such as KIRC, BRCA, CESC, COAD, LGG, LIHC, LUAD, LUSC, PADD, PRAD, and THCA. Besides, TMB, MSI, and neoantigens all reflect the mutation level of organisms contributing to cancer initiation. They are also considered predictors of immunotherapy. We found that in specific cancer types, the *CLEC5A* expression also significantly correlated with TMB, MSI, and neoantigens. Abnormal DNA methylation is a common epigenetic feature of cancer and is associated with the development and progression of the tumor (44, 45). In the present study, *CLEC5A* expression was positively correlated with *DNMT1*, *DNMT2*, *DNMT3*, and *DNMT4* in more than 20 tumors. These results suggested that aberrantly overexpressed CLEC5A plays a vital role in tumorigenesis, which may be related to MMR gene level and DNA methylation.

Tumor immune microenvironment (TIME), an essential part of TME, intimately correlates with tumor immunity accounting for tumor progression (46–48). Immune cells are the main components of TIME (49, 50). Macrophages belong to innate immunity, which is the first defense line for anti-tumor immunity (51–54). The different activation states of macrophages (M1, M2) contribute to anti-tumor and pro-tumor effects, respectively (55, 56). A previous study has revealed CLEC5A as an M2 biomarker. We conducted an immune infiltration analysis. The results implicated that *CLEC5A* expression was positively correlated with immune infiltration levels of macrophages, CAF, and Tregs, whereas it is negatively correlated with MDSC in most cancer types. Tumor purity, a key index reflecting the condition of TME, was indirectly evaluated by the ESTIMATE score, and the distribution of immune and stromal components in TME was reviewed by the immune score and stromal score (57). The present study showed that *CLEC5A* expression positively correlates with an immune score in BLCA, COAD, KICH, LGG, OV, and THCA, with the ESTIMATE score in BLCA, COAD, KICH, LUSC, OV, and READ. These results indicated that CLEC5A might be involved in tumor immunity regulation by mediating immune infiltration. In addition, more than 30 immune checkpoints were positively correlated with *CLEC5A* expression in LGG, OV, and PAAD. The strong correlation between CLEC5A and immune checkpoints suggested that CLEC5A might be an ideal tumor immunotherapeutic target and play specific roles in tumor immunotherapy response and outcome.

It is no doubt that CLEC5A participates in tumorigenesis; the underlying mechanism remains unclear. We implemented the functional enrichment analysis to identify the biological roles of CLEC5A in pan-cancer. The results showed that in addition to activation of EMT and apoptosis and the inhibition of DNA damage response, CLEC5A high expression was mainly related to leukocyte trans-endothelial migration, inflammatory response, and two signaling pathways (IL6-JAK-STAT3 and KRAS signaling). It implicated that CLEC5A is related to tumor initiation and metastasis. Previous studies on dengue virus infection have revealed the role of CLEC5A in disease development and the efficacy of CLEC5A silencing or inhibition in disease treatment (58, 59). Whether CLEC5A can be a target for cancer treatment still needs more experimental exploration. In addition, we revealed potential therapeutic agents by analyzing the correlation between CLEC5A expression and drug sensitivity, which verified the role of CLEC5A as a potential target for tumor treatment.

Even though the pan-cancer analysis covers comprehensive information, this study is still limited. Most results were based on data analysis, and more experimental verification is essential to provide solid evidence. Previous studies have shown that anti-CLEC5A mAb can be applied to treat viral infection with good clinical efficacy. However, whether it can be used as a tumor treatment drug remains to be studied further.

## Conclusions

In summary, we proved that CLEC5A expression is associated with immune infiltration and affects immunotherapy sensitivity patient’s prognosis in pan-cancer, suggesting that CLEC5A can serve as a biomarker for tumor immunity and prognosis of a potential promising anti-tumor therapeutic target.

## Data Availability Statement

The original contributions presented in the study are included in the article/**Supplementary Material**. Further inquiries can be directed to the corresponding authors.

## Author Contributions

WW performed the data analysis and drafted the figures. S-YC wrote the manuscript. ZZL revised the manuscript. RC, QC, and W-JZ conceived and designed the study. Z-PW, ZL, JZ, PL, JY, and LZ supervised the study. All authors approved the submitted version of the manuscript.

## Funding

This study was supported by the National Natural Science Foundation of China (No. 81903663, No. 81803582, No. 81903725) the Hunan Provincial Natural Science Foundation of China (No. 2022JJ20095, No. 2020JJ5944, No. 2020JJ4896), and the Hunan Provincial Health Committee Foundation of China (No. 202204044869).

## Acknowledgments

This work was supported in part by the High Performance Computing Center of Central South University.

## Conflict of Interest

The authors declare that the research was conducted in the absence of any commercial or financial relationships that could be construed as a potential conflict of interest.

## Publisher’s Note

All claims expressed in this article are solely those of the authors and do not necessarily represent those of their affiliated organizations, or those of the publisher, the editors and the reviewers. Any product that may be evaluated in this article, or claim that may be made by its manufacturer, is not guaranteed or endorsed by the publisher.
