# Supplementary material for: A Pan-Cancer Analysis Reveals CLEC5A as a Biomarker for Cancer Immunity and Prognosis
Source: Front Immunol. 2022 Aug 1;13:831542. doi: 10.3389/fimmu.2022.831542 (PMC9376251; doi:10.3389/fimmu.2022.831542)
Supplement: Supplementary file 1 [file DataSheet_1.docx]

**Supplementary Figures**


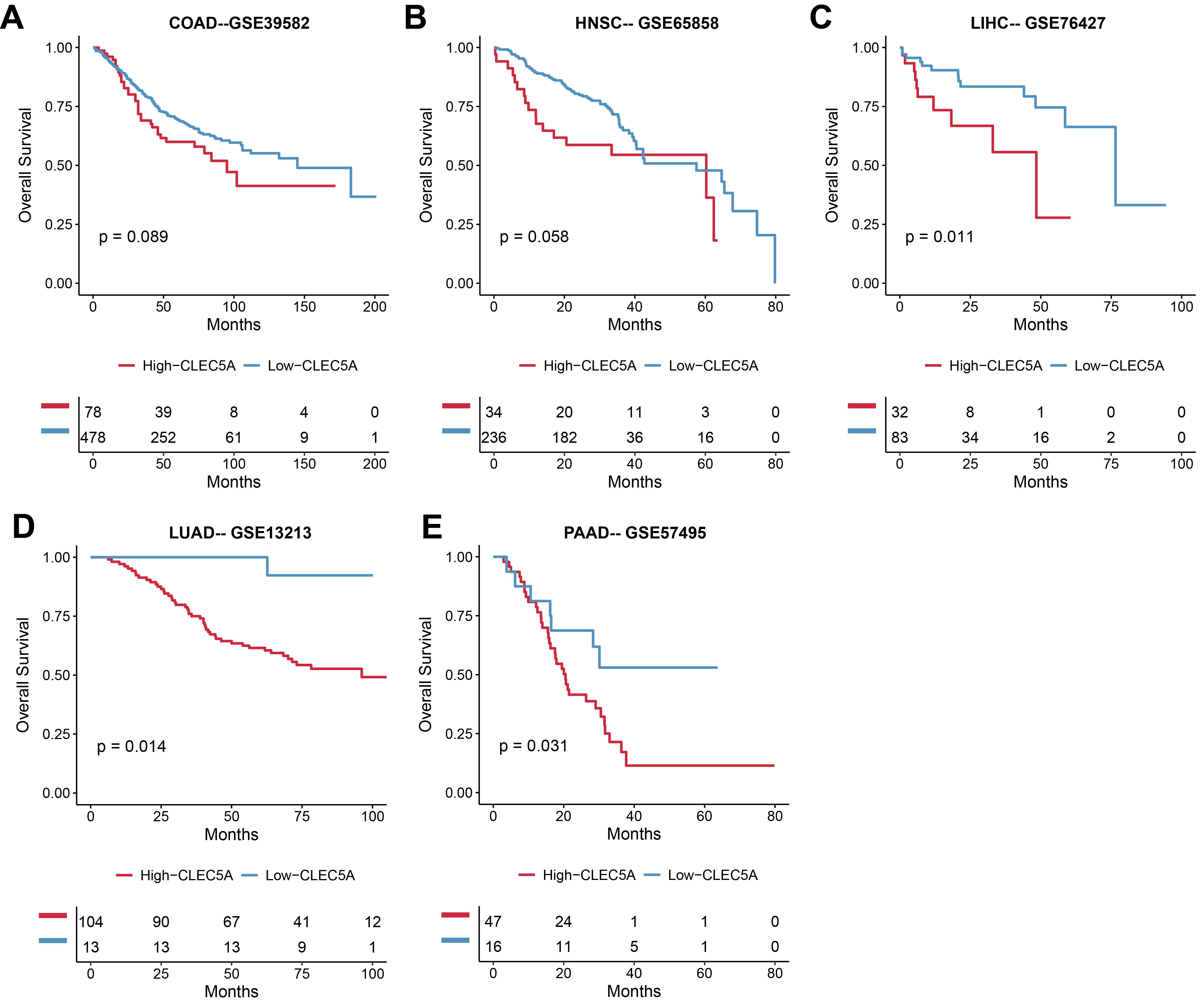


**Supplementary Figure 1.** (A) Kaplan-Meier curves for patient’s OS stratified by different expression levels of *CLEC5A* in COAD with GSE39582. (B) Kaplan-Meier curves for patients’ OS stratified by different expression levels of *CLEC5A* in HINSC with GSE65858. (C) Kaplan-Meier curves for patients’ OS stratified by different expression levels of *CLEC5A* in LIHC with GSE76427. (D) Kaplan-Meier curves for patients’ OS stratified by different expression levels of *CLEC5A* in LUAD with GSE13213. (E) Kaplan-Meier curves for patients’ OS stratified by different expression levels of *CLEC5A* in PAAD with GSE57495.


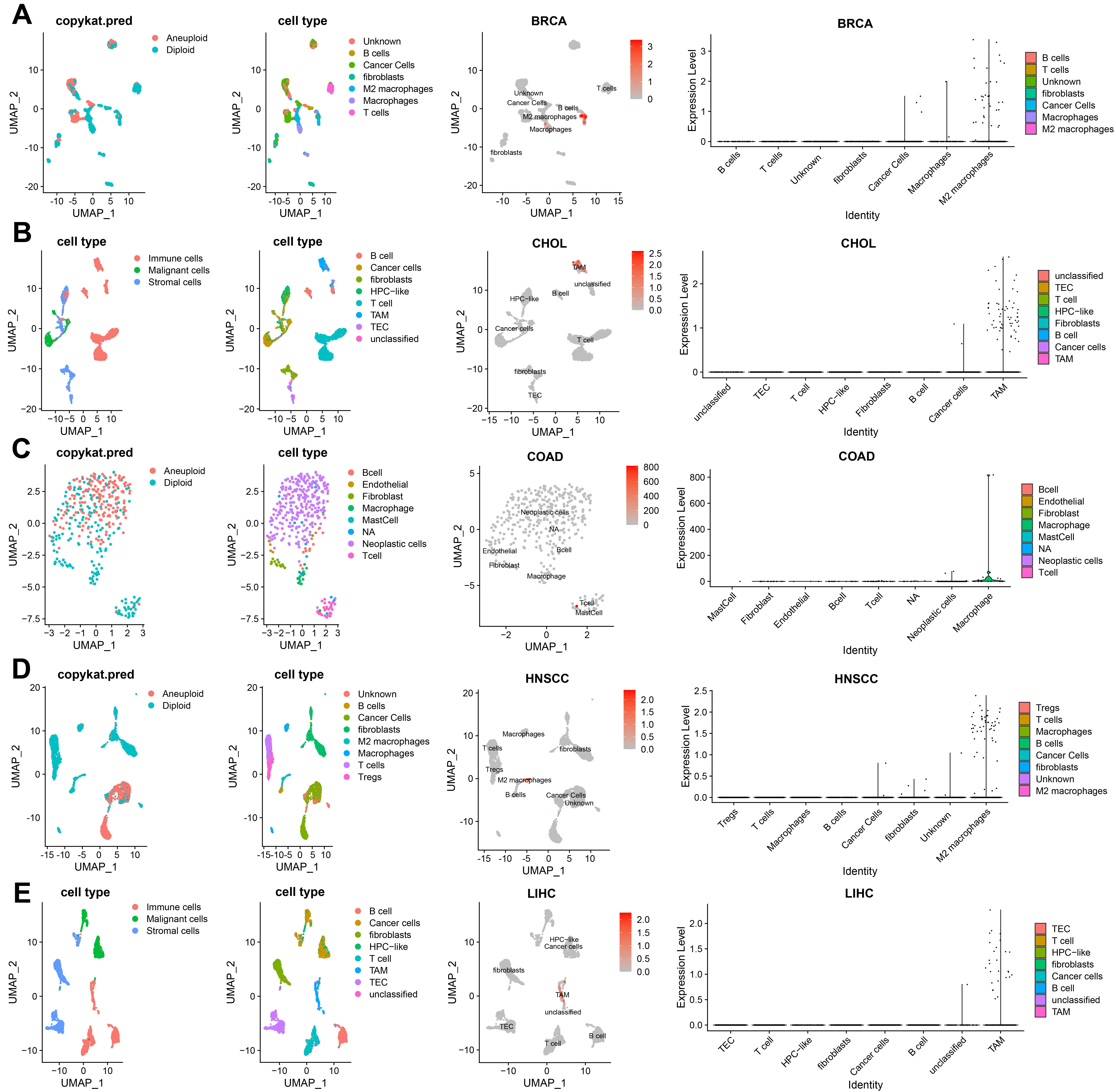


**Supplementary Figure 2. Single Cells analysis of CLEC5A in human cancers**. (A-E) Composition and distribution of single cells and CLEC5A expression in single cells in BRCA, CHOL, COAD, HNSCC, and LIHC. Each cell type is ranked by its average expression value.


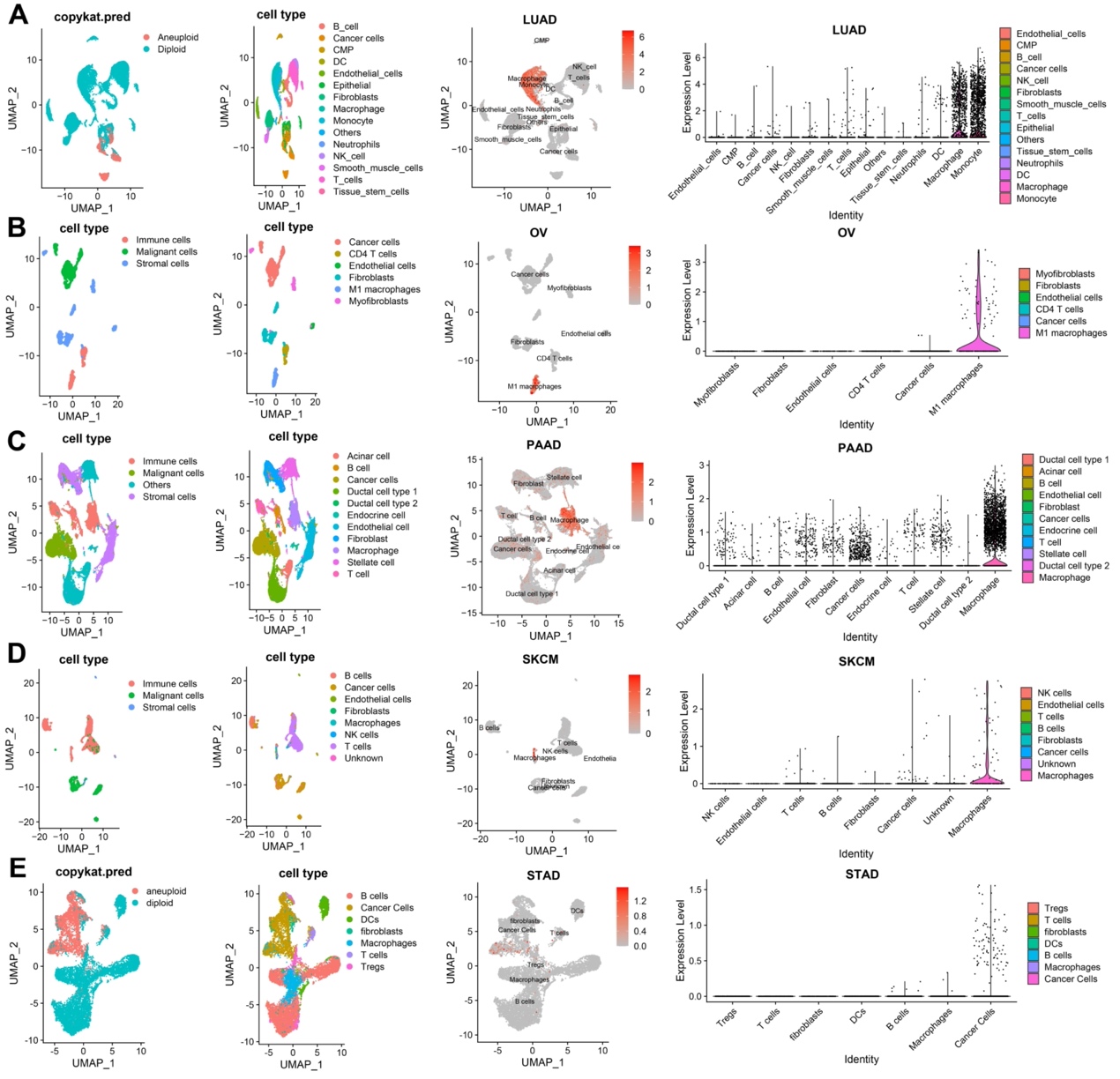


**Supplementary Figure 3. Single Cells analysis of CLEC5A in human cancers**. (A-E) Composition and distribution of single cells and CLEC5A expression in single cells in LUAD OV, PAAD, SKCM, and STAD. Each cell type is ranked by its average expression value.

**
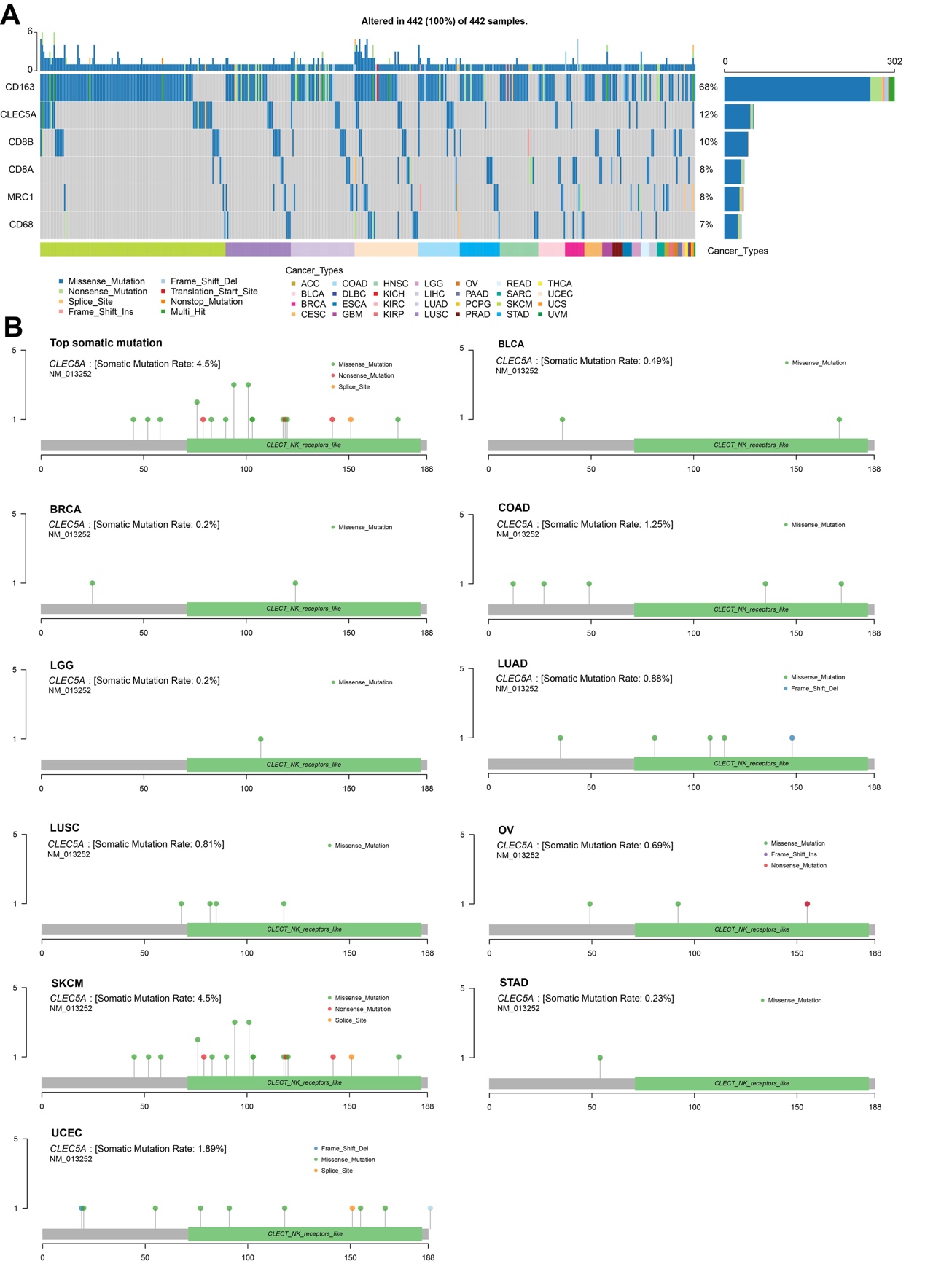
**

**Supplementary Figure 4. *CLEC5A* SNVs in pan-cancer.** (A) SNV profiles of *CLEC5A*, *CD68*, *MRC1*, *CD8A*, *CD8B,* and *CD163* in pan-cancer. Each gray vertical bar represents a patient. The side and top column diagrams show the numbers of variants in each sample or each gene. (B) SNV mutation model in cancers from TCGA database.

**
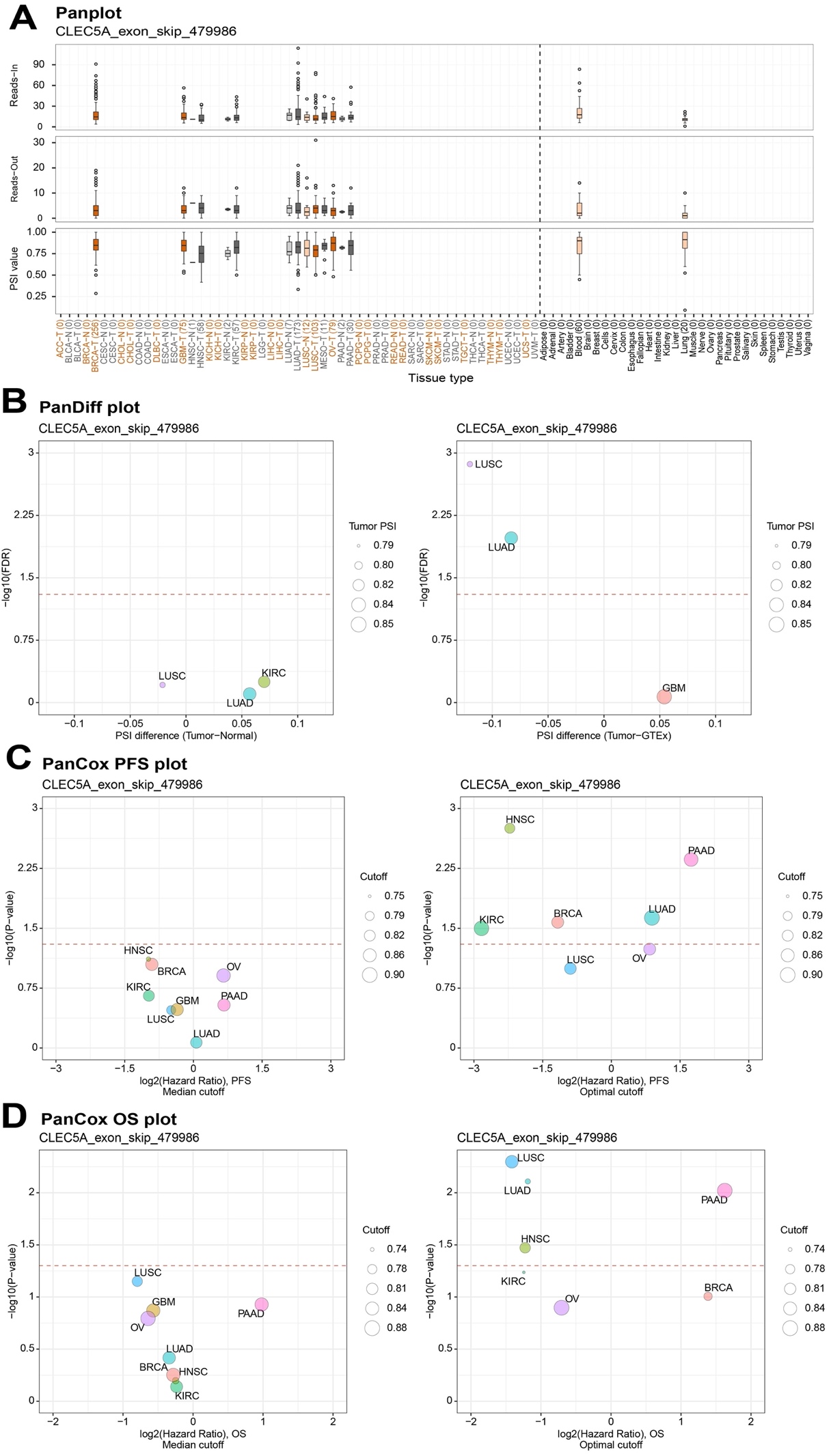
**

**Supplementary Figure 5. *CLEC5A* alternative splicing in human cancers**. (A) PanPlot displayed the percent spliced in (PSI) distribution of alternative splicing events across different TCGA cancers and GTEx tissues. (B) Percent splice in (PSI) differences of the alternative splicing events between TCGA tumor tissue and adjacent normal tissue (left) and GTEx normal tissue (right). Alternative splicing events with absolute delta PSI＞0.1 and Benjamini–Hochberg adjust *p*＜0.05 were considered significant differential alternative splicing events. (C, D) PanCox plot exhibits the correlation between the percent splice in (PSI) distribution of *CLEC5A* alternative splicing events and PFS and OS in TCGA cancers. Alternative splicing events with Log-Rank *p*＜0.05 were considered significant survival-associated alternative splicing events.


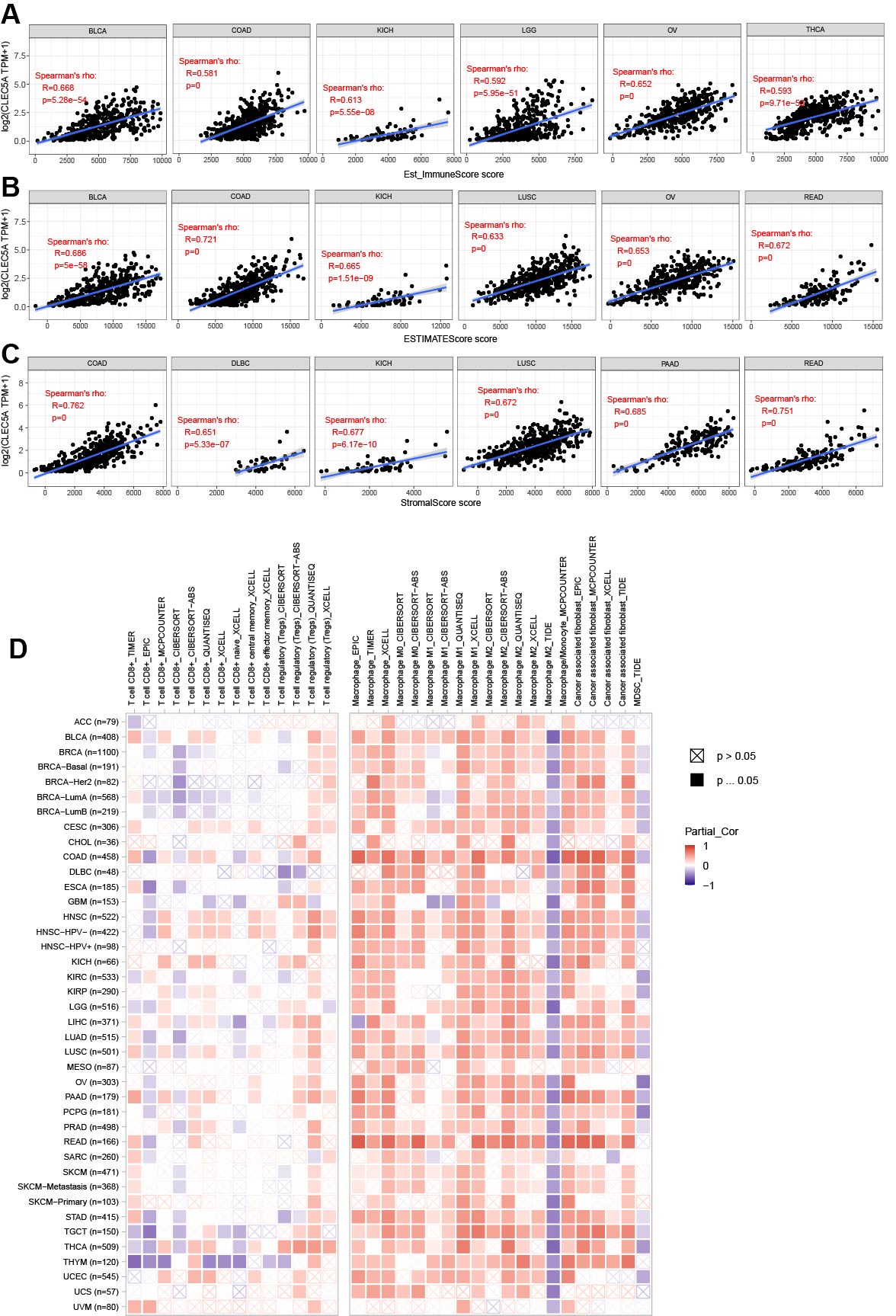


**Supplementary Figure 6. *CLEC5A* expression correlates with immune cell infiltration in human cancers**. (A-C) Correlation between *CLEC5A* expression and immune score, ESTIMATE score, and stromal score in the top 6 cancer types. (D) Correlation between CLEC5A expression and several immune cell infiltration levels in pan-cancer. The color from blue to red represents the correlation level.

**
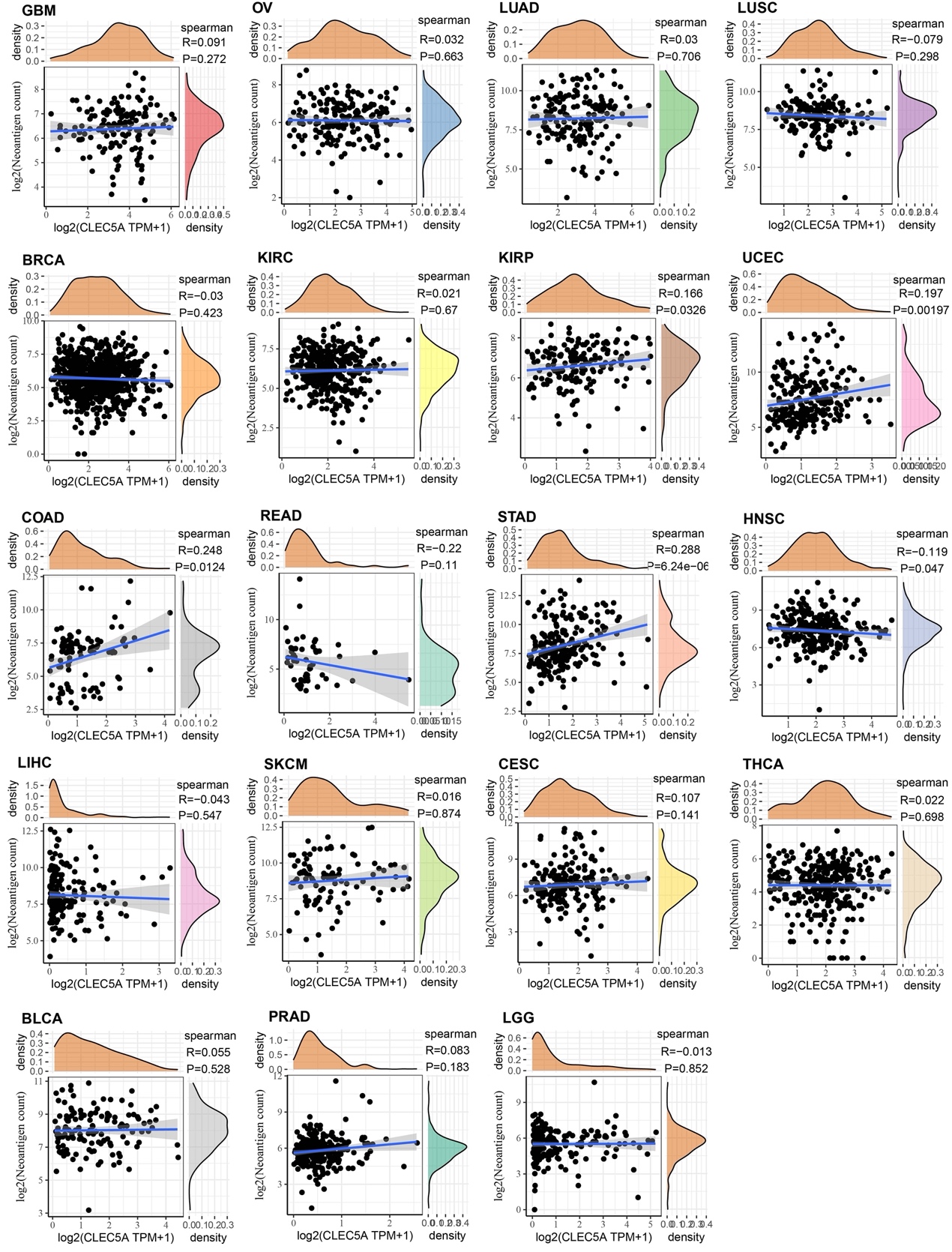
**

**Supplementary Figure 7. Correlation between *CLEC5A* expression and neoantigens in pan-cancer.** *P* < 0.05 means statistical significance.


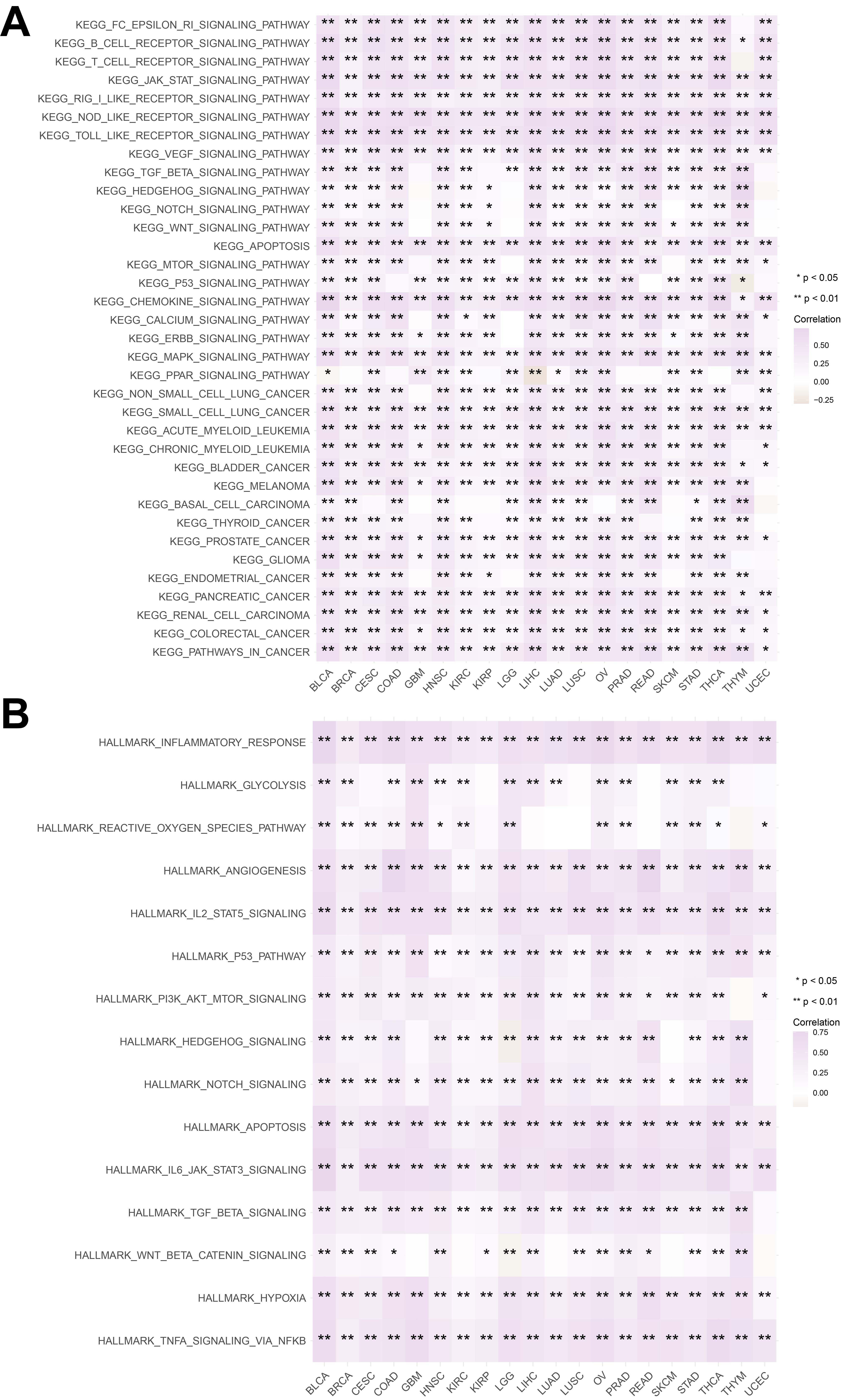


**Supplementary Figure 8.** **KEGG and HALLMARK functional enrichment analysis** **of *CLEC5A*-related patterns** **in pan-cancers.**

**
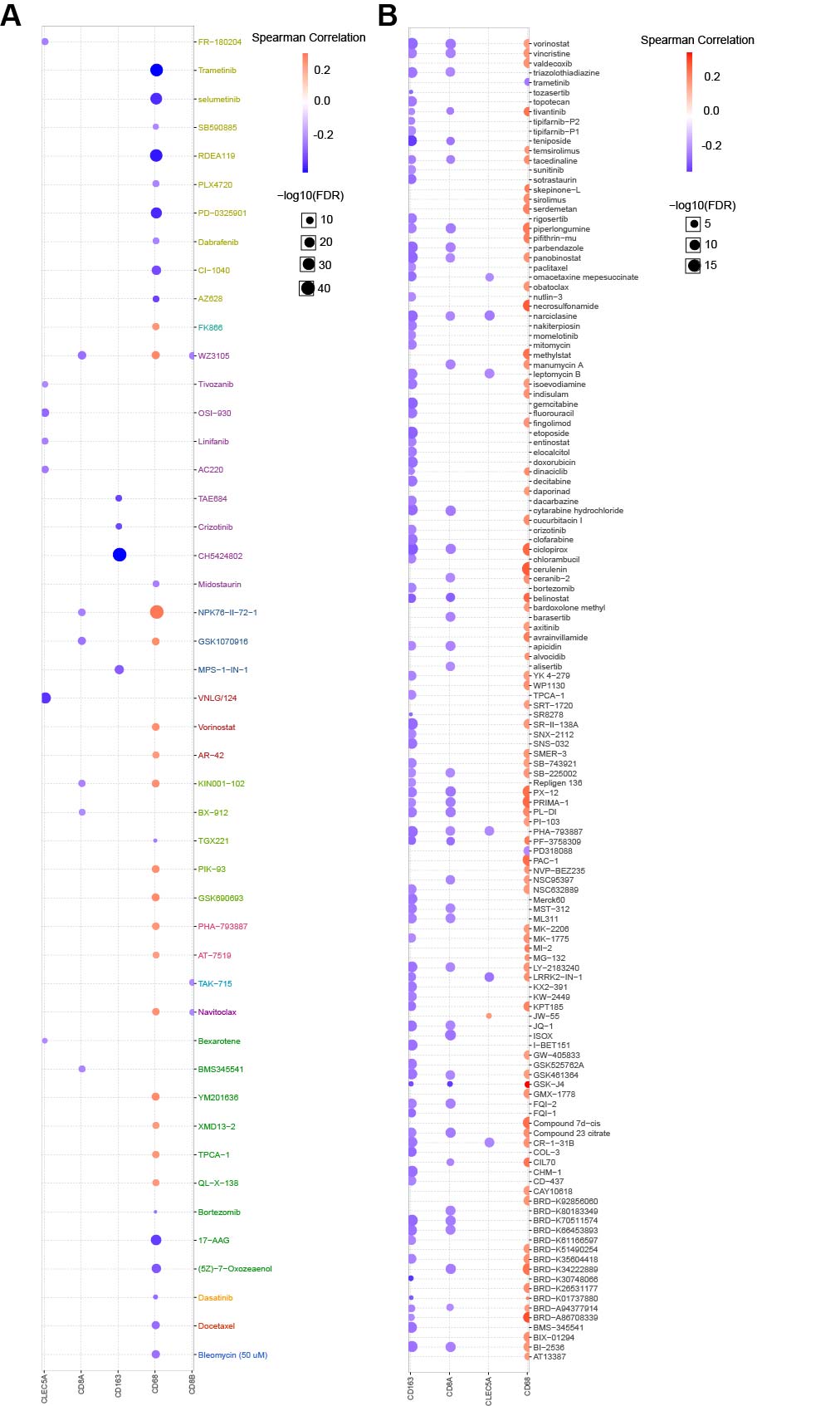
**

**Supplementary Figure 9. Correlation of *CLEC5A* expression with small molecule/drug sensitivity from (A) GDSC database and (B) CTRP database, respectively.**
